# Which resolution?

**DOI:** 10.1107/S205225252300698X

**Published:** 2023-09-01

**Authors:** Colin Nave

**Affiliations:** a Diamond Light Source, Harwell Science and Innovation Campus, Didcot OX11 0DE, United Kingdom; University of Chicago, USA

**Keywords:** resolution, protein crystallography, X-ray imaging, biological cells

## Abstract

The relationship between the contrast to noise ratio and intensity based cross-correlation coefficients for both protein crystallography and X-ray imaging are compared. It is concluded that, for protein crystallography at near atomic resolution, the intensity based cross-correlation coefficients give a reasonable indication of the quality of the corresponding electron density. For X-ray imaging of biological materials such as cells and soft tissue, the wide range of contrast of the features means that intensity based correlation coefficients can give a poor indication of the interpretability of an image. Rather than the term resolution, it is the contrast to noise ratio for a feature of interest at the relevant spatial frequency that is more relevant. Additional metrics are required to describe the quality of an image, and these are discussed.

## Abstract 2

The use of intensity based cross-correlation coefficients between half datasets is compared for near atomic resolution protein structure determination and X-ray imaging of cells and tissues.

## 1. Introduction

It has become common in both protein crystallography and single-particle electron microscopy of proteins to quote a resolution for the data and use a cross-correlation coefficient (CCC) between half datasets to determine this resolution. A threshold beyond which the data merges into the noise can then be determined and the resolution can thus be used to judge the usefulness of the data for a particular purpose. Following this, resolution estimates based on the correlation coefficient have also been adopted for X-ray imaging of cells and biological tissue. However, the range of contrast for such specimens varies much more than for protein structure determination. In these circumstances, the usefulness of a single threshold for estimating the resolution of the data is much more limited.

The present paper does not address the ongoing issue of what the threshold should be for the purpose of a claimed resolution or data cut-off and it does not address the issue of whether any threshold should be a fixed value or information content based. A description of the application of the Fourier shell correlation (FSC) and CC_1/2_ values to obtain resolution estimates in single-particle electron microscopy and protein crystallography, respectively, is given by Dubach & Guskov (2020 ▸).

Many papers acknowledge that any single number is insufficient to describe the interpretability of an image obtained from an instrument. Similarly, it is widely recognized that the term resolution should be regarded as a property of the instrument, defined by parameters such as the aperture of a lens, the size of a focal spot, or the size and distance of a detector for X-ray diffraction/scattering. For an image, the aim is to obtain the desired contrast to noise ratio (CNR) at the relevant spatial frequency for a feature of interest. This is a better although more complex criterion than resolution and emphasizes the visibility of a feature of known dimensions. The relationship between this and the intensity based CCC will depend on the distribution of density for the overall specimen.

The questions being addressed are:

(1) What CNR at a necessary spatial frequency does one need to identify a particular feature in an electron density map with a desired confidence level?

(2) What is the required metric in the intensity data, corresponding to this spatial frequency, to support the interpretation at the desired confidence level?

(3) What additional concise information would be useful to give an estimate of the interpretability of an image?

The above depends on knowledge of the distribution of electron densities as a function of spatial frequency. For the case analysed by Karplus & Diederichs (2015 ▸), a Wilson distribution of intensities was assumed. This is an approximation for the case of protein crystallography and further approximations such as 〈*I*/σ〉 being close to 〈*I*〉/〈σ〉 were made. This allowed the derivation of approximate expressions relating the average signal to noise ratio for the intensities, 〈*I*/σ〉_merged_, to the correlation coefficient CC_1/2_ between half datasets (Karplus & Diederichs, 2015 ▸). Despite the approximations, the comparison between CC_1/2_and 〈*I*/σ〉_merged_ is useful for indicating the relationship between the two most common metrics for defining a resolution.

For the case of X-ray imaging of cells and tissues, a wide range of electron density distributions are possible. If a prior estimate for the electron density distribution can be obtained from similar samples then, in principle, it is possible to go from a desired CNR for a particular feature to a target correlation coefficient at the relevant spatial frequency (FSC and CC_1/2_ are normally plotted as a function of spatial frequency). The procedure for a simple case is described in Section 5. In the other direction, if FSC or CC_1/2_ values are obtained at a particular spatial frequency, it should be possible to estimate the CNR for features of interest. This would cover the case where strong sharp features dominate the distribution, perhaps leaving the features of interest with very poor statistics. An example could be imaging cells at higher energies where high-density features such a polyphosphate bodies and starch granules can dominate over the membrane features which might be of primary interest. Comparison of an image of a biological cell obtained in the water window with one at higher energy shows that the observable features are dramatically different even though the claimed resolution, obtained via correlation coefficient thresholds, might be the same.

## 2. Relationship between intensity correlation coefficients and desired contrast

Ideally one would like to go from a desired CNR for a specific feature at the relevant spatial frequency to a correlation coefficient for intensities at the same spatial frequency. Assumptions and approximations are required for this as listed below.

(i) A prior estimate of the electron density probability distribution at the relevant spatial frequency. This would be affected by the proportion of heavy to light atoms when operating at near atomic resolution or the proportions of condensed chromatin (heterochromatin) to uncondensed chromatin within a biological cell.

(ii) Prior knowledge of any characteristic distances between features. Examples include the repeat distances of helices within proteins/nucleic acids or the bilayer spacing within membranes.

(iii) The distribution of intensities at a relevant spatial frequency. For a repeating sample, as in crystallography, this is obtained from the measured intensity distribution. For an image it is obtained from the Fourier transform of the image. In both cases there will be a measurement error, leading to a correlation coefficient less than 1 between half datasets.

(iv) A model for the measurement errors. This could assume that all errors are derived from Poisson statistics or that the value of sigma is nearly the same for each measurement (applicable where the background is the main source of noise).

### 2.1. Comparison of crystallographic and non-crystallographic cases

For the crystallographic case, measurements normally consist of the integrated intensity of the diffraction spots. This means that no information is obtained concerning the non-repeating parts of the crystal. Differences between adjacent unit cells give short-range disorder resulting in diffuse scatter between the diffraction spots. This diffuse scatter can be the main source of background and, for weak spots, can therefore be the main source of noise in the data. For a non-crystallographic case, the measurements are made over the complete scattering pattern and the material surrounding any feature of interest gives the contrast (ρ_f_ − ρ_s_), where ρ_f_ is the density of the feature and ρ_s_ is the density of the surroundings.

For the non-crystallographic case, where the reconstruction is carried out for the whole object, the corresponding scattered intensities derived from the object boundaries will be strong and influence intensity statistics such as intensity based CCCs. For this reason, a Gaussian or similar smearing is used at the boundary of the object to obtain intensity based CCC values. The distribution of intensities captures both the contrast of individual features and the interference between them due to common interparticle distributions. The FSC formulation (van Heel & Schatz, 2005 ▸) is widely used for single-particle electron microscopy and has been adopted for X-ray imaging.

If a continuous intensity distribution is measured, it is possible to obtain an estimate of the Poisson noise by oversampling. This can complement the estimates obtained for a photon counting detector. However, other sources of error such as sample or instrument instabilities and radiation damage will generally occur.

## 3. Common distributions

The electron density distributions covered below are, in principle, independent of whether the sample is crystallographic or non-crystallographic, with the crystallographic case having a multiplication factor given by the number of unit cells.

### 3.1. Atomic resolution regime

At atomic resolution the Wilson distribution can be applied. In the ideal case, this assumes equal atoms so approximation (i) in Section 2 holds. The random distribution of interatomic distances [approximation (ii)] allows a random phase distribution to be assumed, giving the Wilson distribution of intensities [approximation (iii)]. Finally, at high resolution, the background can be the dominant contribution to the measurement error, allowing the approximation that the same values of sigma can be applied for all reflections satisfying approximation (iv).

Because the electron density distribution is similar for different proteins, there is some merit in quoting a single agreed – though subjective – threshold for the correlation coefficient and calling this the resolution. This is particularly the case for the majority of ordered atoms in the interior of a protein where the scattering factors are similar. One would expect a higher contrast from sulfur atoms and a lower contrast from hydrogen atoms while, at the protein surface, disorder would lower the contrast. For single-particle electron microscopy, this can be handled if desired by the concept of local resolution (*e.g.* Penczek, 2020 ▸) or via *Q* values (Pintilie *et al.*, 2020 ▸). However, what this is really measuring is the decrease in contrast due to the disorder.

### 3.2. General imaging regime

In solution scattering, the particles of interest are separated by a much greater distance than the spatial frequency of interest. This means that interference effects between the particles can be ignored. The one-dimensional distance (or pair) distribution function of a particle is obtained from the scattered intensities without loss of information. It is possible to extend this concept to three dimensions with an approximate model of a biological cell. This only contains features giving a distribution of electron density (positive and negative contrast) at a particular spatial frequency and random distances between the features [satisfying both criteria (i) and (ii)]. The square of the electron density distribution can then be used to give the intensity distribution [criteria (iii)] as inter particle interference does not occur. Finally, if it is assumed that only Poisson noise is present, an estimate can be made for the errors from the square root of the intensities [satisfying criteria (iv)], noting that this is different from the distribution of contrasted electron densities which can be positive or negative.

The relationships discussed above between a target CNR for electron density and a correlation coefficient for intensity will not generally hold. Typical investigations for cellular imaging have the aim of identifying differences between heterochromatin and euchromatin, or identifying the location of virus particles inside the cell. In these cases, the electron density probability distribution in the sample could change significantly. For another example, see Jakubauskas *et al.* (2021 ▸). However, if the probability distribution of electron density at a particular spatial frequency together with a corresponding distribution of scattered intensity both remain stable, then a reference sample can provide useful information linking a desired contrast to noise level to a target CCC for the intensities.

## 4. Contrast to noise ratio as a function of spatial frequency

In general, a feature (*e.g.* an atom, group of atoms, whole protein, virus particle) cannot be defined by a single voxel as any feature has an associated form factor. To accurately represent this in an electron density map, several voxels will be needed for each feature. Taken together, these voxels can be represented by a single ‘Shannon’ sample of the density, centred on the feature, with a form factor corresponding to that for the feature. However, it is assumed for the present purpose that a single feature can be represented by a single voxel centred on the feature, following Howells *et al.* (2009 ▸).

To calculate a defined probability *P* for a false positive identification of a feature, the relevant signal to noise ratio needs to be defined. This depends on the contrast of the feature and the noise level. The contrast can be defined as |ρ_f_ − ρ_s_|, where ρ_f_ is the electron density of the feature at the spatial frequency (*F*) defined by the feature size and ρ_s_ is the density of the surroundings. The ratio |ρ_f_ − ρ_s_|/(σ_f_ + σ_s_)^1/2^, defined as the CNR (*e.g.* Timischl, 2015 ▸), can then be used to avoid a false-positive identification. The CNR is related to the Rose criterion (Rose, 1948 ▸) and can be used to derive the minimum required dose (energy deposited per unit volume) and fluence (incident photons per unit area) for achieving the required CNR. The Rose model emphasizes the contrast for a voxel of a particular size. It is consistent with the resolution defined as the half period of an equal-width line and space grating as used to measure the resolution of an instrument. If two features of size *d* are separated by a gap of size *d* then they it should be possible to identify them if there is adequate contrast at a spatial frequency of 1/(2*d*). Note that the equal-width line and space grating is a poor model for distinguishing individual atoms as most of the electrons are concentrated near the atomic centre, over a distance much less than interatomic distances.

As well as the shot noise, errors from the instrument (*e.g.* beam instabilities) and errors from the model (*e.g.* inability to interpret disordered solvent) can contribute to the CNR for a particular feature. These errors will also contribute to difference maps (*e.g.* *mF*_o_ − *DF*_c_ maps) used in protein crystallography.

In the work by Howells *et al.* (2009 ▸), the requirement for observing a feature above the surroundings with a signal to noise ratio of 5 (Rose criterion of 5) was that the electron density contrast (*e.g.* ρ_f_ − ρ _s_ ) should scatter 25 photons. Although a signal to noise ratio of 5 might seem to be a severe requirement, there can be many voxels in a three-dimensional image, increasing the probability of a false positive. Starodub *et al.* (2008 ▸) suggested that only 6.25 photons would be needed as the signal to noise ratio for the amplitudes (from which the electron densities are derived) follows the square root of the intensities. This apparent discrepancy can be resolved if it is recognized that the probability of a feature being observed falsely above a background is a separate criterion from the accuracy of the electron density for an isolated feature. Only 6.25 photons (standard error 2.5) for the intensity measurement would be required to achieve a standard error of 5 for the electron density of an isolated particle. In any case, there are other options for the noise model in addition to using the amplitude (Godard *et al.*, 2012 ▸).

### 4.1. Contrast for X-ray imaging and protein crystallography

**Figure 1:**
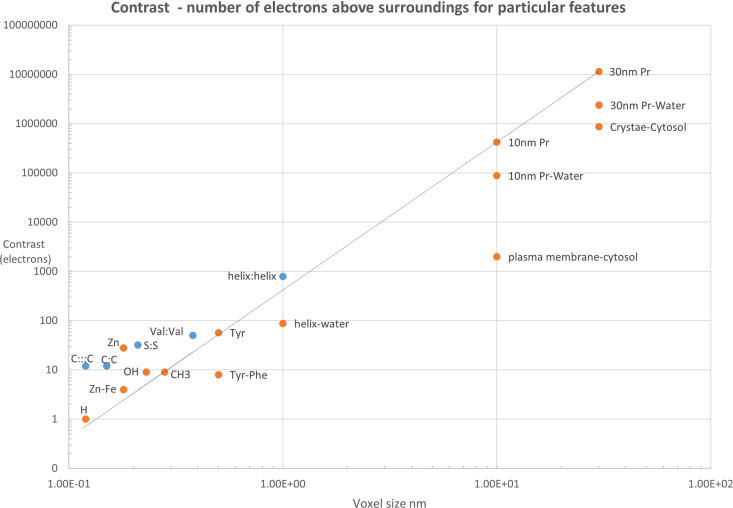
Single features (*e.g.* 10 nm protein, helix exposed to solvent, CH_3_ group, Zn atom) are shown in orange. Paired features (*e.g.* helix:helix, C≡C triple bond) are shown in blue. The contrast (in electrons) for paired features is given by the total number of electrons (*e.g.* 12 electrons for a C:C pair). This could be divided by a factor of 2 if one is interested in observing each component independently. When distinguishing between features (*e.g.* Zn and Fe, Tyr and Phe, or a protein surrounded by water), the difference in the number of electrons is relevant and the minus sign is used (*e.g.* Zn–Fe). For single features, the voxel size is given by the cube root of the atomic, group or molecular volume (as stated in Section 4, the atomic volume is a rather poor approximation for deriving the electron density distribution of an atom). For paired features it is given by the distance between the individual features. The straight line gives the contrast for different size proteins in a vacuum. Density values for cellular components are adapted from the work by Nave (2018 ▸). See Table S1 of the supporting information from which all values were derived.

Fig. 1 ▸ illustrates the contrast for the case of some features in a biological cell and for a protein at near atomic resolution.

For protein crystallography, most work is carried out at spatial frequencies where van der Waals and covalent bonding distances apply. In these circumstances, the contrast for a particular feature is relative to vacuum rather than disordered or unresolved solvent molecules. The electron density distribution and the intensity distribution as a function of spatial frequency is reasonably well defined by Wilson statistics. The concept of contrast then comes in when distinguishing between features with small differences in the number of electrons (*e.g.* different metal atoms, distinguishing between carbon, nitro­gen or oxygen in a substrate). A special procedure (CC_anom_) has been developed for the case of small changes due to anomalous scattering (Karplus & Diederichs, 2015 ▸). For a given value of CC_1/2_, features below the line in Fig. 1 ▸ will have a lower contrast compared with features above.

At spatial frequencies common in X-ray imaging, the concept of contrast is more important. This is because there are a wide range of different features, some of which have densities well above the surrounding solvent (*e.g.* starch granules, polyphosphate bodies) and some of which can be almost contrast matched (*e.g.* plasma membranes contrasted against cytosol as shown in Fig. 1 ▸) or have negative contrast (*e.g.* lipid droplets). Specifying a resolution for a particular image (*e.g.* via some correlation coefficient threshold or making measurements across some boundary) demonstrates that a particular instrument is capable of reaching this resolution. It does not mean that any feature of a particular size can be reliably identified.

## 5. Relationships between contrast to noise ratio and cross correlation coefficients for a model cell

To illustrate the process of linking the desired CNR with CC_1/2_, a cartoon biological cell is constructed with a set of features with a different contrast, different size and different probability (*P*_f_) of occurring. The contrast for a particular feature (ρ_fc_) can be defined as ρ_f_ − ρ_cytosol_ where ρ_f_ is the electron density of the feature and ρ_cytosol_ is the electron density of the surrounding cytosol. The contrast can also be defined as ρ_f_ − 〈ρ〉, where 〈ρ〉 is the average density. The latter definition of the contrast is used in the example below. The size of the voxel (*d*_v_) is chosen and the calculation is made for the distribution of the total scattered X-rays from each voxel. In the calculation below, the corresponding value of CC_1/2_ therefore corresponds to the total intensity distribution from voxels of a defined size. The calculation could then be repeated with different sized voxels to obtain an estimate of CC_1/2_ for different spatial frequencies.

The following relationship between CC_1/2_ values and other intensity statistics is derived in the appendix of Karplus & Diederichs (2012 ▸).

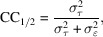

where σ_τ_ is the standard deviation of the true distribution of intensities and σ_ɛ_ denotes the mean error within a half dataset. Assuming Poisson statistics for the scattering of each feature, 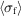 replaces the σ derived from merging statistics in Box1 of Karplus & Diederichs (2015 ▸), with σ_f_ being the standard deviation of photons scattered by a feature. This gives 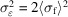 .

The number of electrons (*N*_ef_) in the feature above or below the average (or above or below the cytosol) is given by 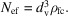 This is reduced for features smaller than the voxel size by a factor corresponding to the difference in volume. For example, the plasma membrane (10 nm thick) has a volume of 30 × 30 × 10 nm within a 30 nm voxel.

To obtain an estimate for the distribution of scattered photons, it is assumed that each feature is randomly positioned in the cell so the features can be considered to scatter independently. This assumption is an approximation for a real sample. The assumption means that the scattered intensity from each feature is proportional to the square of the contrast. This gives 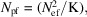 where *N*_pf_ is the number of photons scattered by the feature, *N*_ef_ is the number of electrons in the feature above or below the average (or above or below the cytosol), and K is a constant of proportionality with a value dependent on the X-ray exposure.

The above applies for the case where the intensities are derived from the real space image. For the case where the intensities are obtained directly from the scattered X-rays, an additional term is needed, corresponding to the Lorentz factor in crystallography, as uniform sampling of the intensities does not occur in tomographic imaging. This affects the intensities and the errors if Poisson statistics is assumed. It also means that the required fluence and dose follows the fourth power of the resolution rather than the third power which would otherwise be obtained from the volume of the feature. This is discussed in detail by Gureyev *et al.* (2018 ▸).

The average value of *N*_p_ is given by

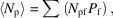

and the value of σ_τ_ is given by

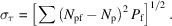

Assuming a perfect instrument and following Howells *et al.* (2009 ▸), σ_f_ should follow the square root of *N*_pf_ giving

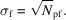

The average value of σ_f_ is given by

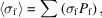

and the required value of 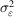 is

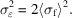

Table 1 ▸ gives the predicted value for CC_1/2_ for a biological cell containing seven components at a voxel size *d*_v_ of 30 nm and an X-ray exposure corresponding to *K* = 1.33 × 10^11^.

With the exposure and corresponding CC_1/2_ value the 30 nm protein will scatter 17 photons (*N*_pf_ value) above the surroundings giving the probability of a false identification of approximately 0.1% (depending on the exact distribution). The probability of a false identification will be higher for the 10 nm protein. It will also be much higher for the plasma membrane unless one is able to correlate the contrast across several voxels.

Contrast can be improved by heavy atom stains, and this is commonly used for imaging of brain tissue where optimizing the stain density is often necessary for obtaining the required information. Measurements of stain density can be found in the work by Fera *et al.* (2020 ▸). An analysis similar to that given in Table 1 ▸ might prove useful for both optimizing and monitoring stain density for a particular system. It is possible that stained material will have a much simpler electron density distribution meaning that, as for protein structure determination, the use of the term resolution will have some merit.

## 6. Conclusions

Although the use of the term resolution could be considered a largely terminological issue, in the case of X-ray imaging of cells and tissue the use of the term is more misleading than illuminating.

Given the limitations of a single resolution estimate for an image, several additional metrics could be calculated from the images or measured/derived intensities. In order to be useful, they should be either single numbers or one-dimensional graphs. Possibilities include a complete FSC or CC_1/2_ graph as a function of spatial frequency; intensity distributions at selected spatial frequencies; histograms of image electron densities or refractive indices, for both the real and the imaginary components, at selected spatial frequencies; calculating the entropy from the histogram of densities at selected spatial frequencies. Note however that this does ignore the spatial distribution of densities in the image.

A discussion is required regarding which would be most useful. This could occur under existing initiatives such REMBI as described by Sarkans *et al.* (2021 ▸).

Even for protein structure determination the established methods for determining a resolution are still under debate. The use of a single metric such as resolution for defining the quality of an electron density map is potentially misleading. It would be of some benefit if a second easily understood metric could be agreed or at least error estimates for the claimed resolution are provided. The problem for both X-ray imaging and protein structure determination is that any metrics must be reasonably robust, agreed by the experts and understandable by less-expert users.

Finally, compiling a list of electron densities as a function of spatial resolution for typical cells would be a useful exercise for X-ray imaging.

## Supplementary Material

Table S1 to accompany Fig. 1. DOI: 10.1107/S205225252300698X/mf5065sup1.pdf

## Figures and Tables

**Table 1:** Parameters for a cartoon biological cell to illustrate the effect of observing features with different contrast, size and probability of occurring rather than representing a real cell which would have a much wider range of features The calculations are carried out for a voxel size of 30 nm, corresponding to a spatial frequency of 0.0167 nm^−1^ and a *K* value of 1.3 × 10^11^.

Feature	Cytosol	Plasma membrane	10 nm protein	Cristae	30 nm protein	Hetero-chromatin	Lipid droplet
Type	Globular	Planar	Globular	Planar	Globular	Globular	Globular
*P*_f_	0.45	0.10	0.20	0.05	0.05	0.10	0.05
*d*_f_ (nm)	30	10	10	28	30	30	30
ρ_f_ (e nm^−3^)	340	350	420	380	420	380	300
ρ_fc_ (e nm^−3^)	−25	−15	55	15	55	15	−65
*N*_ef_	−6.75 × 10^5^	−1.35 × 10^5^	5.50 × 10^4^	3.78 × 10^5^	1.49 × 10^6^	4.05 × 10^5^	−1.76 × 10^6^
*N* ^2^_ef_	4.56 × 10^11^	1.821 × 10^10^	3.03 × 10^9^	1.43 × 10^11^	2.21 × 10^12^	1.64 × 10^11^	3.08 × 10^12^
|*N*_ef_|*P*_f_	3.04 × 10^5^	1.35 × 10^4^	1.10 × 10^4^	1.89 × 10^4^	7.43 × 10^4^	4.05 × 10^4^	8.78 × 10^4^
*N* ^2^_ef_ *P*_f_	2.05 × 10^11^	1.82 × 10^9^	6.05 × 10^8^	7.14 × 10^9^	1.10 × 10^11^	1.64 × 10^10^	1.54 × 10^11^
*N*_pf_	3.50	0.14	0.023	1.10	17.0	1.26	23.7
*N*_pf_ *P*_f_	1.58	0.014	0.00465	0.055	0.848	0.126	1.18
*N* ^2^_pf_	12.3	0.0197	5.41 × 10^−4^	1.21	288	1.59	561
*N* ^2^_pf_ *P*_f_	5.53	0.00197	1.08 × 10^−4^	0.0604	14.4	0.159	28.1
σ_f_	1.87	0.374	0.153	1.05	4.12	1.12	4.87
σ_f_ *P*_f_	0.842	0.0374	0.0305	0.0524	0.206	0.112	0.243
ρ_f_ *P*_f_	153	35	84	19	21	38	15

Whole cell							
〈ρ〉	365						
〈N_p_〉	3.81						
*d*σ_τ_	6.54						
〈σ_f_〉^2^	2.32						
σ_ε_^2^	4.65						
CC_1/2_	0.902						
