# Supplementary material for: Which resolution?
Source: IUCrJ. 2023 Sep 1;10(Pt 5):603–9. doi: 10.1107/S205225252300698X (PMC10478518; doi:10.1107/S205225252300698X)
Supplement: Supplementary file 1 [file m-10-00603-sup1.pdf]

IUCrJ

Volume 10 (2023)

Supporting information for article:

Which resolution?

Colin Nave

S1. Table to accompany fig. 1.

Feature label	Feature	Total excess number electrons	Voxel size (nm)
C:C	carbon:carbon	12	0.15
C:::C	carbon:carbon triple	12	0.12
S:S	sulphur:sulphur	32	0.21
Val:Val	Val:Val sidechains	50	0.38
helix:helix	α helix:α helix, 1 nm length	790	1.00
H	Hydrogen	1	0.12
CH3	Methyl	9	0.28
OH	hydroxyl	9	0.23
Tyr	Tyr	57	0.5
Tyr-Phe	Distinguish Tyr from Phe	8	0.5
Zn	Zn^{++}	28	0.18
Zn-Fe	distinguish Zn^{++} and Fe^{++}	4	0.18
helix-water	α helix – water, 1 nm length	88	1.0
10nm Pr	protein in vacuum	4.2×10^5	10
10nm Pr-Water	protein in water	8.8×10^4	10
Crystae-Cytosol	Crystae in cytosol	8.6×10^5	30
plasma membrane-cytosol	1:1 protein: phospholipid in cytosol	2000	10
30nm Pr	protein in vacuum	1.1×10^7	30
30nm Pr-Water	protein in water	2.4×10^6	30
